# The serum heart-type fatty acid-binding protein (HFABP) levels can be used to detect the presence of acute kidney injury on admission in patients admitted to the non-surgical intensive care unit

**DOI:** 10.1186/s12872-016-0340-1

**Published:** 2016-09-05

**Authors:** Akihiro Shirakabe, Nobuaki Kobayashi, Noritake Hata, Takuro Shinada, Kazunori Tomita, Masafumi Tsurumi, Hirotake Okazaki, Masato Matsushita, Yoshiya Yamamoto, Shinya Yokoyama, Kuniya Asai, Wataru Shimizu

**Affiliations:** 1 Division of Intensive Care Unit, Nippon Medical School Chiba Hokusoh Hospital, 1715 Kamagari, Inzai, Chiba 270-1694 Japan; 2 Department of Cardiovascular Medicine, Nippon Medical School, Tokyo, Japan

**Keywords:** Biomarker, Renal dysfunction, Cardiovascular disease, Emergency care, Mortality

## Abstract

**Background:**

No cardiac biomarkers for detecting acute kidney injury (AKI) on admission in non-surgical intensive care patients have been reported. The aim of the present study is to elucidate the role of cardiac biomarkers for quickly identifying the presence of AKI on admission.

**Methods:**

Data for 1183 patients who underwent the measurement of cardiac biomarkers, including the serum heart-type fatty acid-binding protein (s-HFABP) level, in the emergency department were screened, and 494 non-surgical intensive care patients were enrolled in this study. Based on the RIFLE classification, which was the ratio of the serum creatinine value recorded on admission to the baseline creatinine value, the patients were assigned to a no-AKI (*n* = 349) or AKI (Class R [*n* = 83], Class I [*n* = 36] and Class F [*n* = 26]) group on admission. We evaluated the diagnostic value of the s-H-FABP level for detecting AKI and Class I/F. The mid-term prognosis, as all-cause death within 180 days, was also evaluated.

**Results:**

The s-H-FABP levels were significantly higher in the Class F (79.2 [29.9 to 200.3] ng/mL) than in the Class I (41.5 [16.7 to 71.6] ng/mL), the Class R (21.1 [10.2 to 47.9] ng/mL), and no-AKI patients (8.8 [5.4 to 17.7] ng/mL). The most predictive values for detecting AKI were Q2 (odds ratio [OR]: 3.743; 95 % confidence interval [CI]: 1.693–8.274), Q3 (OR: 9.427; 95 % CI: 4.124–21.548), and Q4 (OR: 28.000; 95 % CI: 11.245–69.720), while those for Class I/F were Q3 (OR: 5.155; 95 % CI: 1.030–25.790) and Q4 (OR: 22.978; 95 % CI: 4.814–109.668). The s-HFABP level demonstrating an optimal balance between sensitivity and specificity (70.3 and 72.8 %, respectively; area under the curve: 0.774; 95 % CI: 0.728–0.819) was 15.7 ng/mL for AKI and 20.7 ng/mL for Class I/F (71.0 and 83.1 %, respectively; area under the curve: 0.818; 95 % CI: 0.763–0.873). The prognosis was significantly poorer in the high serum HFABP with AKI group than in the other groups.

**Conclusions:**

The s-H-FABP level is an effective biomarker for detecting AKI in non-surgical intensive care patients.

## Background

The risk, injury, failure, loss, and end-stage (RIFLE) criteria have been established as a standard method for evaluating acute kidney injury (AKI) in intensive care patients [1, 2]. In a previous study, we reported that patients with AKI, particularly those with a Class I or F status, exhibit worse in-hospital mortality rates and long-term prognoses than no-AKI patients among subjects with acute heart failure (AHF) [3, 4]. Furthermore, 33.2 % of AHF patients already have AKI upon admission to the intensive care unit (ICU) [3], which is associated with a poor in-hospital mortality rate and long-term prognosis [5]. The presence of AKI on admission and a Class I or F status are important factors in AHF patients; therefore, these findings are also important among overall non-surgical intensive care patients. In clinical situations, the detection of AKI on admission, especially a Class F status, is an immediate and essential problem in non-surgical intensive care patients. The timely diagnosis of AKI might propitiate early adjustment of the diuretic dose or justify the use of other drugs as well as the transient early use of renal replacement therapies.

In recent years, authors have found the presence of renal tubular injury to be the mechanism underlying the development of AKI, as determined based on the levels of urinary biomarkers, such as neutrophil gelatinase-associated lipocalin (NGAL), liver fatty acid-binding protein (LFABP) and N acetyl-β-D glucosaminidase (NAG), in patients with contrast media-induced nephropathy or a history of cardiac surgery and those receiving intensive care [6–11]. In several studies, including some meta-analyses, the measurement of NGAL was found to be particularly useful for detecting AKI in non-surgical intensive care patients [12–14]. However, cardiac dysfunction induces renal impairment and organ disorders, and thus these pathologies are comorbidities among non-surgical intensive care patients. It is therefore reasonable to detect the presence of AKI using cardiac biomarkers.

The release of heart-type fatty acid-binding protein (HFABP) following myocardial injury peaks after 4 to 6 h, after which the levels return to baseline within 20 h. Therefore, myocardial injury can be detected relatively early using the HFABP level. We hypothesized that the serum HFABP level is an effective cardiac biomarker for quickly identifying the presence of AKI on admission and investigated the diagnostic value of the serum HFABP level obtained on admission for detecting AKI, particularly a Class I/F status, according to the etiology of the disease among non-surgical intensive care patients.

The aim of present study is to elucidate the role of serum HFABP for quickly identifying the presence of AKI, especially severe criteria of AKI, on admission in non-surgical intensive care patients.

## Methods

### Subjects

**Fig. 1:**
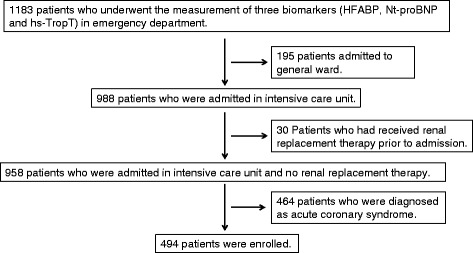
The patient selection process. HFABP, heart-type fatty acid-binding protein; Nt-proBNP, N-terminal pro-brain-type natriuretic peptide; hs-TropT, high-sensitivity troponin-T

We screened 1183 patients who underwent measurement of three biomarkers (HFABP, N-terminal pro-brain-type natriuretic peptide [Nt-proBNP], and high-sensitivity troponin-T [hs-TropT]) in the emergency department at Chiba Hokusoh Hospital, Nippon Medical School, Japan, between March 2011 and November 2013. We excluded patients who were admitted to the general ward, had received renal replacement therapy prior to admission, or were diagnosed with acute coronary syndrome (ACS) (e.g. acute myocardial infarction [AMI] and unstable angina). As a result, 494 patients admitted to the ICU were enrolled in this study. (Fig. 1). Patients with the following diseases were included in the present study: AHF, acute aortic dissection, pulmonary embolism, arrhythmia, Takotsubo cardiomyopathy, coronary spasm angina, infectious disease (sepsis, infective endocarditis, pneumonia, pericarditis, or myocarditis), and emergent respiratory disease.

### Serum biomarker measurement

The blood samples were collected in tubes within 10 min of admission and centrifuged within 30 min after admission. The serum levels of hs-TropT, Nt-proBNP, and HFABP were measured additionally using standard laboratory parameters via standard enzymatic methods: an electrochemiluminescence immunoassay (Roche Diagnostics Ltd, Rotkreuz, Switzerland), a chemiluminescent immunoassay (CLEIA), and a MARKIT-M HFABP enzyme-linked immunosorbent assay kit or LIBLIA H-FABP latex agglutination turbidimetric immunoassay (DS Pharma Biomedical, Osaka, Japan). The measurements of these biomarkers were obtained in all 1183 patients. The lower limit of detection for the serum concentration of HFABP was 1.0 ng/mL, while that of hs-TropT was 3 ng/L.

### Evaluation of AKI

We evaluated the presence of AKI on admission, using only the creatinine criteria of the RIFLE classification [2]. The RIFLE classification is based on the ratio of the serum creatinine value recorded on admission to the baseline creatinine value. The patients were classified as having either no AKI or Class R (risk), Class I (injury), or Class F (failure) AKI.

The serum creatinine levels in the patients without chronic kidney disease (CKD) (diagnosed based on their medical data) were calculated using the Modification of Diet in Renal Disease (MDRD) equation, as recommended by the Acute Dialysis Quality Initiative, by solving the MDRD equation for serum creatinine (CrMDRD), assuming a glomerular filtration rate (GFR) of 75 mL/min/1.73 m^2^ [15, 16]. The baseline level of creatinine was the lowest value recorded during admission in patients with CKD. The lowest of the creatinine values observed during hospitalization or the CrMDRD creatinine level served as the baseline value in patients without CKD.

CKD was diagnosed based on the creatinine value observed within one year. Among patients in whom the creatinine value was not measured within one year before admission, those who had been previously diagnosed with CKD in the past were considered to have CKD. CKD was defined as a low GFR (<60 mL/min/1.73 m^2^) for more than 3 months [17]. Patients who did not have any medical records at Chiba Hokusoh Hospital for the three months prior to admission were diagnosed with CKD using the data obtained at other institutions for the three-month period before admission. Kidney damage, as identified based on abnormal findings on urine and imaging tests [17], was diagnosed in some patients in the present study; therefore, CKD was diagnosed only according to a low GFR with a history exceeding three months. In the present study, 211 of 494 patients (42.7 %) were diagnosed with CKD, including 144 patients (68.2 %) in whom the creatinine value had been measured within the past year and 67 patients (31.8 %) with a medical history of CKD.

### Procedures and prognoses

The occurrence of AKI was evaluated according to the RIFLE classification on admission. No AKI was present in 349 patients (no-AKI), while AKI was detected on admission in 145 patients; the number of patients according to class was as follows: Class R (*n* = 83), Class I (*n* = 26), and Class F (*n* = 36). The serum cardiac biomarker levels were compared between four groups (no-AKI, Class R, Class I, and Class F). The non-surgical intensive care patients were also assigned to four groups according to the quartile of the serum HFABP level: Q1 (*n* = 124), Q2 (*n* = 123), Q3 (*n* = 124) and Q4 (*n* = 123). The independent factors for detecting AKI and a Class F status were selected in a univariate analysis for inclusion in the multivariate logistic regression model.

Receiver-operating characteristic (ROC) curves for the serum biomarkers were calculated to predict the optimal cut-off values, and the sensitivity, specificity, and area under the ROC curve (AUC) were determined in order to identify the optimal values for predicting AKI and Class F patients among the overall patients. The cut-off values were decided by the Youden Method.

The mid-term prognosis, as all-cause death within 180 days, was also evaluated. The patients were clinically followed up at a routine outpatient clinic. The prognoses for patients who were followed up at other institutes were determined via telephone interview. The patients were assigned to other groups based on the cut-off values of the ROC curves for the HFABP level and presence of AKI. The prognostic value of the serum HFABP level and presence of AKI compared with that observed in the low HFABP and no-AKI group as the referent was assessed using a Cox regression hazard model. A Cox regression analysis was performed to obtain the hazard ratio (HR) for 180-day mortality, and the survival rates were analyzed using Kaplan-Meier curves for each biomarker.

### Statistical analysis

All of the data were statistically analyzed using the SPSS 22.0 J software program (SPSS Japan Institute, Tokyo, Japan). All of the numerical data are expressed as the mean ± standard deviation or median (range or 25–75 % interquartile range) by the normality, which was assessed using the Shapiro-Wilk W test. The unpaired Student’s *t-*test or the Mann-Whitney *U*-test were used to compare two groups, and a one-way analysis of variance or the Kruskal-Wallis test were used to compare three or four groups. The comparisons of all proportions were made using a chi-square analysis for two groups and Pearson’s bivariate test for three or four groups.

The significant biomarkers indicating AKI and a Class I/F status on admission were determined using a multivariate logistic regression model. ROC curves were calculated to predict the cut-off values, and the sensitivity, specificity, and area under the curve (AUC) were determined. The cut-off values were determined using the Youden Method. A *P*-value of < 0.05 was considered to be statistically significant.

The prognostic value of the serum HFABP level in the low HFABP and AKI group, high HFABP and no-AKI group, and high HFABP and AKI group compared with that observed in the low HFABP and no-AKI group as the referent was assessed using a Cox regression hazard model. A Cox regression analysis was performed to obtain the HR for 180-day mortality. Thereafter, a multivariate analysis was performed using the variables with a *P*-value of <0.05 in the univariate analysis to examine their independent associations with the 180-day mortality. The survival rates were analyzed between the groups assigned based on the cut-off value of the ROC curve for the HFABP level and classified as having or not having AKI according to a Kaplan-Meier curve, and significant differences were calculated using the log-rank test.

## Results

### Patient characteristics

The differences in the patients’ characteristics according to the RIFLE criteria are shown in Table 1. The patient cohort was 68.0 % male subjects, with a median age of 71 years. The number of arrhythmia and infectious disease patients, lactate levels, serum BUN levels, and serum CRP levels were significantly higher and the systolic blood pressure (BP) values, pH levels, and serum hemoglobin levels significantly lower in the AKI group than in the no-AKI group. In addition, the hs-TropT levels and Nt-proBNP levels were also significantly higher in the AKI group than in the no-AKI group.

**Table 1:** Patient characteristics and RIFLE criteria

	total (*n* = 494)	no AKI (*n* = 349)	AKI			
			Class R (*n* = 83)	Class I (*n* = 36)	Class F (*n* = 26)	*P* value
Age (years old)	71 (62–79)	71 (62–80)	71 (64–79)	67 (62–76)	78 (69–81)	0.092
Gender (male, %)	336 (68.0 %)	237 (67.9 %)	56 (67.5 %)	28 (77.8 %)	15 (57.7 %)	
Etiology						
Acute Heart Failure (yes)	242 (49.0 %)	186 (53.3 %)	37 (44.6 %)	12 (33.3 %)	7 (26.9 %)	0.008
Acute Aortic Dissection (yes)	48 (9.7 %)	34 (9.7 %)	11 (13.3 %)	3 (8.3 %)	0 (0.0 %)	0.255
Pulmonary Thromboembolism (yes)	21 (4.3 %)	17 (4.9 %)	3 (3.6 %)	1 (2.8 %)	0 (0.0 %)	0.624
Arrhythmia (yes)	65 (13.2 %)	38 (10.9 %)	14 (16.9 %)	7 (19.4 %)	6 (23.1 %)	0.109
Coronary Spasms (yes)	22 (4.5 %)	21 (6.0 %)	1 (1.2 %)	0 (0.0 %)	0 (0.0 %)	0.073
Takotsubo Cardiomyopathy (yes)	13 (2.6 %)	10 (2.9 %)	1 (1.2 %)	1 (2.8 %)	1 (3.4 %)	0.829
Infectious disease (yes)	32 (6.5 %)	9 (2.6 %)	9 (10.8 %)	7 (19.4 %)	7 (26.9 %)	<0.001
Other Intensive Care disease (yes)	36 (7.3 %)	20 (5.7 %)	6 (7.2 %)	5 (13.9 %)	5 (19.2 %)	0.028
Past medical history						
Chronic Kidney Disease (yes)	211 (42.7 %)	159 (45.6 %)	31 (37.3 %)	12 (33.3 %)	9 (34.6 %)	0.249
Hypertension (yes)	360 (72.9 %)	260 (74.5 %)	55 (66.3 %)	25 (69.4 %)	20 (76.9 %)	0.435
Diabetes mellitus (yes)	174 (35.2 %)	115 (33.0 %)	36 (43.4 %)	9 (25.0 %)	14 (53.8 %)	0.032
Dyslipidemia (yes)	234 (47.4 %)	170 (48.7 %)	40 (48.2 %)	14 (38.9 %)	10 (38.5 %)	0.544
Vital signs and status						
Systolic blood pressure (mmHg)	147 (116–174)	156 (129–182)	137 (101–164)	115 (103–133)	107 (87–128)	<0.001
Diastolic blood pressure (mmHg)	80 (64–100)	84 (70–101)	74 (60–91)	64 (52–74)	55 (44–70)	<0.001
Pulse Rate (beats/min)	98 (75–119)	100 (78–120)	92 (67–115)	97 (66–110)	83 (59–120)	0.141
Respiratory Rate (beats/min)	25 (20–32)	25 (20–32)	24 (17–34)	21 (18–27)	20 (15–26)	0.050
Body Temperature (°C)	36.3 (35.7–36.8)	36.3 (35.8–36.7)	36.3 (35.5–36.8)	36.7 (36.2–37.3)	36.6 (35.9–37.5)	0.006
Body Mass Index (%)	22.9 (20.3–25.4)	23.1 (20.4–25.8)	23.0 (21.0–25.2)	21.7 (19.8–23.8)	22.7 (19.0–25.1)	0.094
LVEF (%)	50 (32–65)	50 (35–64)	47 (29–65)	45 (29–69)	50 (45–65)	0.466
Arterial blood gas						
pH	7.40 (7.28–7.44)	7.40 (7.29–7.44)	7.37 (7.20–7.42)	7.40 (7.31–7.46)	7.35 (7.27–7.42)	0.052
PCO_2_ (mmHg)	38 (33–48)	39 (34–48)	36 (33–53)	32 (27–40)	34 (30–43)	<0.001
PO_2_ (mmHg)	101 (73–156)	100 (72–149)	112 (73–181)	116 (80–162)	81 (68–128)	0.249
HCO_3_ - (mmol/l)	22.4 (19.2–24.9)	23.1 (20.6–25.1)	20.4 (17.8–24.3)	20.2 (15.5–23.5)	18.8 (14.7–23.0)	<0.001
SaO_2_ (%)	97 (94–99)	97 (94–99)	97 (94–99)	98 (96–99)	96 (93–98)	0.243
Lactate (mmol/l)	1.7 (1.1–3.3)	1.5 (1.1–2.6)	2.7 (1.4–6.1)	2.0 (1.1–8.4)	2.4 (1.2–7.4)	<0.001
Laboratory data						
WBC (U/l)	9,630 (7,000–12,590)	9,245 (6,868–12,018)	10,250 (7,915–13,630)	10,800 (7,810–14,710)	8,370 (6,678–13,405)	0.018
Hemoglobin (g/dl)	12.6 (10.7–14.3)	12.7 (11.1–14.4)	13.4 (10.8–14.7)	11.3 (9.4–12.7)	9.7 (8.5–11.9)	<0.001
BUN (mg/dl)	22.4 (16.5–33.3)	19.9 (15.9–28.2)	24.9 (18.3–33.2)	39.3 (23.8–51.1)	55.6 (46.5–83.1)	<0.001
Creatinine (mg/dl)	1.10 (0.82–1.69)	0.99 (0.75–1.35)	1.23 (1.00–1.53)	1.74 (1.12–2.16)	3.27 (2.20–4.84)	<0.001
Sodium (mmol/l)	140 (137–142)	140 (138–142)	139 (136–142)	137 (133–142)	139 (136–142)	0.007
Potassium (mmol/l)	4.1 (3.8-4.7)	4.0 (3.7-4.5)	4.3 (3.9-4.9)	4.5 (3.8-5.5)	4.9 (3.9-6.5)	<0.001
BS (mg/dl)	157 (122–235)	154 (119–233)	174 (144–303)	143 (124–242)	130 (97–174)	<0.001
CRP (mg/dl)	0.61 (0.11–3.17)	0.41 (0.09–1.27)	0.99 (0.14–5.88)	3.34 (0.83–9.08)	3.51 (0.42–8.17)	<0.001
Uric Acid (mg/dl)	6.8 (5.2–8.2)	6.4 (5.1–7.6)	8.2 (6.1–9.2)	7.4 (6.7–9.9)	8.4 (6.0–11.8)	<0.001
BNP (pg/ml)	431 (95–951)	426 (76–901)	389 (157–750)	691 (139–2007)	437 (263–1231)	0.090
hs-TropT (ng/ml)	0.05 (0.02–0.12)	0.04 (0.02–0.09)	0.05 (0.02–0.18)	0.07 (0.05–0.38)	0.08 (0.05–0.14)	<0.001
Nt-proBNP (pg/ml)	2,550 (633–8,653)	2,333 (378–6,753)	2,746 (924–7,958)	6,975 (1,946–47,228)	10,959 (2,686–23,400)	<0.001
H-FABP (ng/ml)	11.7 (6.2–27.8)	8.8 (5.4–17.7)	21.1 (10.2–47.9)	41.5 (16.7–71.6)	79.2 (29.9–200.3)	<0.001
Mechanical Support (cases) during the ICU stay						
NPPV (yes, %)	209 (42.3 %)	161 (46.1 %)	35 (42.2 %)	10 (27.8 %)	3 (11.5 %)	0.002
ETI (yes, %)	121 (24.5 %)	53 (15.2 %)	36 (43.4 %)	18 (50.0 %)	14 (53.8 %)	<0.001
Pacing (yes, %)	37 (7.5 %)	20 (5.7 %)	8 (9.6 %)	6 (16.7 %)	3 (11.5 %)	0.069
IABP (yes, %)	26 (5.3 %)	9 (2.6 %)	10 (12.0 %)	5 (13.9 %)	2 (7.7 %)	<0.001
PCPS (yes, %)	17 (3.4 %)	5 (1.4 %)	7 (8.4 %)	3 (8.3 %)	2 (7.7 %)	0.002
CHDF (yes, %)	60 (12.1 %)	15 (4.3 %)	11 (13.3 %)	17 (47.2 %)	17 (65.4 %)	<0.001

*LVEF* left ventricular ejection fraction measured on echocardiography, *WBC* white blood cell, *BUN* blood urea nitrogen, *BS* blood sugar, *CRP* C-reactive protein, *BNP* brain natriuretic peptide, *hs-TropT* high-sensitivity troponin T, *Nt-proBNP* N-Terminal pro-brain-type natriuretic peptide, *H-FABP* heart-type fatty acid binding, *ICU* intensive care unit, *NPPV* noninvasive positive pressure ventilation, *ETI* endotracheal intubation, *IABP* intra-aortic balloon pumping, *PCPS* percutaneous cardiopulmonary support, *CHDF* continuous hemodiafiltration

*p* value between the three groups determined according to a one-way analysis of variance or the Kruskal-Wallis test

### HFABP and AKI

**Fig. 2:**
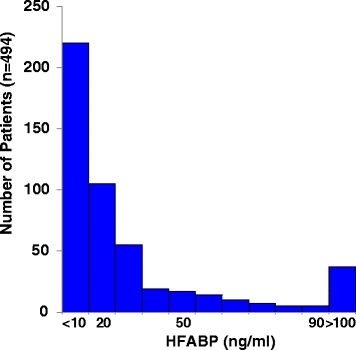
The distribution of the HFABP levels. The median value among all 494 intensive care patients was 11.7 ng/ml. The HFABP level was <10 ng/mL in 220 patients (44.5 %) and >100 ng/mL in 37 patients (7.5 %). HFABP, heart-type fatty acid-binding protein

The distribution of the HFABP levels in the overall non-surgical intensive care patients is described in Fig. 2. Among all 494 patients admitted to receive non-surgical intensive care, the HFABP levels were <10 ng/mL in 220 patients (44.5 %) and >100 ng/ml in 37 patients (7.5 %). The median (range) HFABP levels were significantly higher in the Class F (79.2 [29.9 to 200.3] ng/mL) than in the Class I (41.5 [16.7 to 71.6] ng/mL), the Class R (21.1 [10.2 to 47.9] ng/mL), and the no-AKI (8.8 [5.4 to 17.7] ng/mL) groups. (Table 1).

The incidence of AKI and in-hospital mortality by quartile of HFABP level is noted in Table 2. The rate of patients classified into the no-AKI, Class R, Class I, and Class F groups was significantly different among the quartile groups. The in-hospital mortality was poorer in the Q4 group than in the Q3, Q2, and Q1 groups and in the Q3 group than in the Q2 and Q1 groups.

**Table 2:** Relationships between the quartiles of HFABP and the incidence of AKI, levels of cardiac markers and in-hospital mortality

	Q1	Q2	Q3	Q4	
	H-FABP≦6.2 (*n* = 124)	6.3≦H-FABP≦11.6 (*n* = 123)	11.7≦H-FABP≦27.8 (*n* = 124)	27.9≦H-FABP (*n* = 123)	*p* value
Acute kidney injury					
no-AKI (yes, %)	113 (91.1 %)	102 (82.9 %)	90 (72.6 %)	44 (35.8 %)	<0.001
Class R (yes, %)	9 (7.3 %)	16 (13.0 %)	23 (18.5 %)	35 (28.5 %)	<0.001
Class I (yes, %)	1 (0.8 %)	5 (4.1 %)	6 (4.8 %)	24 (19.5 %)	<0.001
Class F (yes, %)	1 (0.8 %)	0 (0.0 %)	5 (4.0 %)	20 (16.3 %)	<0.001
Biomarkers					
hs-TropT (ng/ml)	0.02 (0.01–0.03)	0.04 (0.02–0.06)	0.06 (0.03–0.10)	0.14 (0.06–0.54)	<0.001
Nt-proBNP (pg/ml)	351 (68–1,748)	2,401 (925–5,451)	5,739 (1,991–13,267)	7929 (2,164–23,269)	<0.001
In-hospital mortality					
dead (yes, %)	3 (2.4 %)	5 (4.1 %)	18 (14.5 %)	38 (30.9 %)	<0.001

*H-FABP* heart-type fatty acid binding, *LVEF* left ventricular ejection fraction measured on echocardiography; *WBC* white blood cell; *BUN* blood urea nitrogen, *BS* blood sugar, *CRP* C-reactive protein, *BNP* brain natriuretic peptide, *hs-TropT* high-sensitivity troponin T, *Nt-proBNP* N-Terminal pro-brain-type natriuretic peptide, *hs-CRP* high-sensitivity C-reactive protein, *NPPV* noninvasive positive pressure ventilation, *ETI* endotracheal intubation, *IABP* intra-aortic balloon pumping, *PCPS* percutaneous cardiopulmonary support, *CHDF* continuous hemodiafiltration, *ICU* intensive care unit

*p* value between the quartiles of H-FABP determined using a variance analysis and the Kruskal-Wallis test

The results of the multivariate logistic regression analysis for detecting the presence of AKI showed the specific biomarkers to be Q2 (odds ratio [OR]: 3.743; 95 % confidence interval [CI]: 1.693–8.274, *p* = 0.001), Q3 (OR: 9.427; 95 % CI: 4.124–21.548, *p* < 0.001) and Q4 (OR: 28.000; 95 % CI: 11.245–69.720, *p* < 0.001), while the markers indicating a Class I/F status were Q3 (OR: 5.155; 95 % CI: 1.030–25.790, *p* = 0.047) and Q4 (OR: 22.978; 95 % CI: 4.814–109.668, *p* < 0.001) (Table 3).

**Table 3:** Multivariate analysis of the associations between acute kidney injury and the clinical findings

	AKI						Class I/F					
	Univariate analysis			Multivariate analysis			Univariate analysis			Multivariate analysis		
	OR	95 % CI	*p* value	OR	95 % CI	*p* value	OR	95 % CI	*p* value	OR	95 % CI	*p* value
Quartiles of H-FABP												
Q1 (H-FABP≦6.2)	1.000			1.000			1.000			1.000		
Q2 (6.3≦H-FABP≦11.6)	2.453	1.095– 5.498	0.029	3.743	1.693–8.274	0.001	2.585	0.492–13.584	0.262	3.194	0.582–17.523	0.181
Q3 (11.7≦H-FABP≦27.8)	5.030	2.311–10.947	<0.001	9.427	4.124–21.548	<0.001	5.938	1.288–27.374	0.022	5.155	1.030–25.790	0.046
Q4 (27.9≦H-FABP)	12.778	5.774 –28.777	<0.001	28.000	11.245–69.720	<0.001	33.975	8.009–144.122	<0.001	22.978	4.814 –109.668	<0.001
Adjusting Factors												
Chronic kidney disease	1.196	0.786–1.819	0.404				0.652	0.373–1.141	0.134			
Age (≧71 years old)	0.921	0.626–1.356	0.677				0.887	0.520–1.512	0.659			
MBP (≧102 mmHg)	0.260	0.171–0.393	<0.001	0.264	0.172–0.442	<0.001	0.140	0.067–0.290	<0.001	0.191	0.087–0.416	<0.001
LVEF (≧51 %)	0.757	0.493–1.163	0.204				0.533	0.486–1.453	0.840			
Hemoglobin (≧12.7 g/dl)	0.681	0.461–1.006	0.053				0.259	0.138–0.483	<0.001	0.430	0.212–0.874	0.020
Hs-TropT (≧0.048 ng/ml)	2.319	1.555–3.458	<0.001	0.736	0.428–1.265	0.267	4.035	2.160–7.536	<0.001	1.220	0.553–2.691	0.623
Nt-ProBNP (≧2553 pg/ml)	1.665	1.126–2.462	0.011	0.824	0.497–1.366	0.452	2.325	1.322–4.091	0.003	1.023	0.500–2.094	0.950

*HR* hazard ratio, *CI* confidence interval, *H-FABP* heart-type fatty acid binding, *MBP*mean blood pressure, *LVEF* left ventricular ejection fraction measured on echocardiography, *Hs-TropT* high-sensitivity troponin T, *Nt-proBNP* N-Terminal pro-brain-type natriuretic peptide

### Diagnostic value of the H-FABP level

**Fig. 3:**
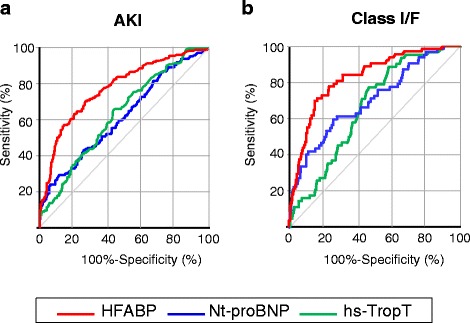
**a** The sensitivity and specificity of the HFABP level for detecting AKI were 70.3 and 77.4 % (AUC: 0.774; 95 % CI: 0.728–0.819), respectively, with a cut-off value of 15.7 ng/mL. **b** The sensitivity and specificity of the HFABP level for detecting a Class I/F status were 71.0 and 83.1 % (AUC: 0.818; 95 % CI: 0.763–0.873), respectively, with a cut-off value of 29.2 ng/mL for the overall patients. HFABP, heart-type fatty acid-binding protein; AKI, acute kidney injury; AUC, area under the receiver-operating characteristic curve

The ROC curves are shown in Fig. 3. The s-HFABP level demonstrating an optimal balance between sensitivity and specificity for detecting the presence of AKI (70.3 and 72.8 %, respectively; AUC: 0.774; 95 % CI: 0.728–0.819) was 15.7 ng/mL for the overall patients, while that for the Nt-proBNP level was 13,829 ng/L (29.0 and 88.8 %, respectively; AUC: 0.618; 95 % CI: 0.562–0.673), and that for the hs-TropT level 48 ng/L was (64.8 and 56.3 %; AUC: 0.622; 95 % CI: 0.570–0.675) (Fig. 3-a). With respect to indicating a Class I/F status, the optimum levels for sensitivity and specificity were 29.2 ng/mL for the HFABP level (71.0 and 83.1 %, respectively; AUC: 0.818; 95 % CI: 0.763–0.873), 6583 ng/L for the Nt-proBNP level (59.7 and 73.4 %, respectively; AUC: 0.691; 95 % CI: 0.616–0.766), and 48 ng/L for the hs-TropT level (77.4 and 54.1 %, respectively; AUC: 0.666; 95 % CI: 0.604–0.728) (Fig. 3-b).

### Prognosis

**Fig. 4:**
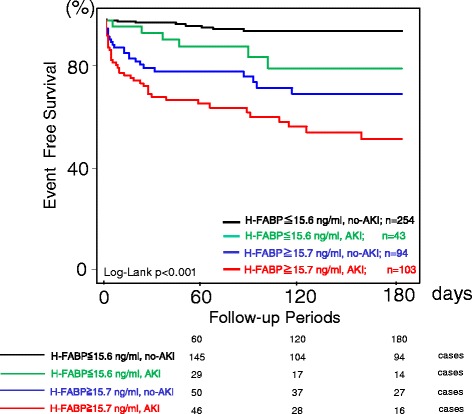
The Kaplan-Meier survival curves showed that the prognosis, including all-cause death, was significantly poorer in the high serum HFABP (≥15.7 ng/mL) with AKI group than in the low serum HFABP (<15.7 ng/mL) with AKI group, high serum HFABP without AKI group, and low serum HFABP without AKI group. HFABP, heart-type fatty acid-binding protein; AKI, acute kidney injury

The multivariate Cox regression model indicated that an HFABP level of ≥15.7 ng/mL and the presence of AKI (HR: 11.593, 95 % CI: 5.154–26.078; *p* < 0.001) were independent predictors of 180-day mortality (Table 4). The Kaplan-Meier survival curves showed that the prognosis, including all-cause death, was significantly poorer in the high serum HFABP with AKI group than in the high serum HFABP without AKI group, low serum HFABP with AKI group, and the low serum HFABP without AKI group (Fig. 4).

**Table 4:** Cox regression analysis of the associations between 180-day cumulative mortality and the clinical findings

	Univariate analysis			Multivariate analysis		
180-days mortality	HR	95 % CI	*p* value	HR	95 % CI	*p* value
H-FABP level and AKI status						
H-FABP≦15.6 and no-AKI	1.000			1.000		
H-FABP≦15.6 and AKI	4.128	1.432–11.898	0.009	2.676	0.920–7.783	0.071
H-FABP≧15.7 and no-AKI	8.070	3.592–18.131	<0.001	9.268	3.835–22.396	<0.001
H-FABP≧15.7 and AKI	14.037	6.556–30.054	<0.001	11.593	5.154–26.078	<0.001
Adjusting factors						
Chronic kiedney disease	0.887	0.560–1.405	0.609			
Age (≧69 years old)	1.036	0.659–1.629	0.879			
MBP (≧99 mmHg)	0.205	0.114–0.366	<0.001	0.239	0.130–0.441	<0.001
LVEF (≧51 %)	0.652	0.396–1.074	0.093			
Hemoglobin (≧13.5 g/dl)	0.567	0.352–0.912	0.019	0.990	0.603–1.624	0.968
Hs-TropT (≧0.064 ng/ml)	2.429	1.478–3.994	<0.001	0.818	0.469 –1.424	0.477
Nt-ProBNP (≧930 pg/ml)	1.598	1.001–2.552	0.050			

*HR* hazard ratio, *CI* confidence interval, *H-FABP* heart-type fatty acid binding, *MBP* mean blood pressure, *LVEF* left ventricular ejection fraction measured on echocardiography, *Hs-TropT* high-sensitivity troponin T, *Nt-proBNP* N-Terminal pro-brain-type natriuretic peptide

## Discussion

The serum levels of HFABP were elevated in non-surgical intensive care patients. An elevated HFABP level may be useful for detecting the presence of AKI, especially a Class I/F status.

### Serum HFABP levels in ACS patients

We first evaluated all of the patients in the intensive care unit, including ACS patients, and investigated the efficacy of the serum HFABP level for detecting AKI. However, the diagnostic value, as represented by the AUC, was not adequate among the overall patients. After narrowing down the study population to the patients in present study, excluding ACS patients, the diagnostic value increased. In almost all of the ACS patients, the serum HFABP levels were elevated due to the presence of immediate and excessive myocardial damage induced by myocardial ischemia. HFABP is abundant in the cytosol and easily released into the circulation when the cardiomyocyte membrane is injured [18]. Therefore, measuring the HFABP level is a mainstay for early diagnosis of ACS, and this parameter is used as the gold standard biomarker for detecting ACS in the early phase [19–22]. The majority of previous reports about the serum HFABP have focused on ACS patients. Therefore, ACS, an emergent ischemic heart condition, is an independent factor for an increased serum HFABP level. In practice, the serum levels of HFABP were markedly higher in the ACS patients than in the other etiology groups in the present study, and some ACS patients with no-AKI were included in the Q3 and Q4 groups. These results suggest that patients with ACS should be excluded when discussing the incidence of AKI.

### Serum HFABP levels by disease

The efficacy of the HFABP level except for in ACS patients was first reported in HF patients. In these previous reports, the HFABP level exhibited superior sensitivity to the troponin T level for detecting ongoing myocardial damage [23] and was reported to be a sensitive marker of minor myocardial injury in patients with congestive HF [24]. Furthermore, the serum HFABP level indicates the presence of latent and ongoing cardiomyocyte damage; therefore, it is a sensitive marker of minor myocardial injury in patients with congestive HF [25].

Seino et al. first reported that some patients with acute aortic dissection and pulmonary embolism also exhibit positive test results for HFABP and that 40 % of patients with non-cardiovascular conditions have an elevated HFABP level [25]. The authors speculated the reason for this finding to be the result of diminished renal clearance [26] and assumed that minor myocardial damage associated with acute hemodynamic deterioration leads to an elevated HFABP level. Hazui et al. also demonstrated that patients with acute aortic dissection have elevated HFABP levels [27]. HFABP is present in skeletal muscle tissue; therefore, HFABP is released into the blood from skeletal muscle rather than cardiac muscle, and the authors speculated that serum HFABP might be derived from the aortic wall. The HFABP level has also been reported to be effective for risk stratification in the setting of pulmonary embolism [28, 29]. Among patients with pulmonary embolism, the HFABP levels may be elevated as a consequence of right ventricular wall stress due to a highly elevated right ventricular afterload.

However, Jo et al. reported that the serum HFABP concentrations are frequently elevated in patients with severe sepsis and septic shock [30]. Because patients with sepsis or septic shock tend to exhibit multiple organ failure, including AKI, and since HFABP is eliminated via renal clearance, the concentrations of HFABP may be increased in subjects with a decreased renal function [31]. In addition, during severe sepsis and septic shock, the catabolism of glycogen and lipids increases the levels of free fatty acids, which may directly lead to an elevated HFABP concentration [32]. Furthermore, septic injury induces metabolic lipid disorders and the release of free radicals, resulting in damage to the vital organs and increased HFABP levels [32]. Such sequelae would of course be related to sepsis-induced myocardial dysfunction.

### Mechanisms for detecting AKI by serum HFABP levels

The mechanisms underlying an increased HFABP level might differ based on the ACS situation in intensive care patients. As stated above, the release of HFABP is postulated in various hypotheses, and minor myocardial injury would be major reason for an increased HFABP level. The leakage of small amounts of macromolecules can be caused by mechanical stretching mechanisms, such as ischemic stress induced by non-cardiogenic shock, which would lead to the release of HFABP into the blood. However, minor myocardial injury alone may not adequately explain the elevation of the HFABP levels observed in non-cardiac patients. A reduction in the amount of skeletal muscle tissue [32] and increase in the free acids level by the catabolism of glycogen [32] could also induce an elevation of the H-FABP level. Furthermore, lipid disorders and the release of free radicals would also lead to increased HFABP levels [32]. Vital organ functions are damaged in almost all non-surgical intensive care patients in various situations, which may result in an increased HFABP level. Therefore, the degree of leakage of HFABP may be greater in severe-condition intensive care patients who have suffered multiple organ failure and vital organ damage. Given that AKI is a component of multiple organ failure, serum HFABP level might be increased in AKI patients due precisely to the multiple organ failure. Serum HFABP is also excreted by renal tubular cells; therefore, patients with an acutely diminished renal function—and thus AKI—are unable to clear large amounts of HFABP. These proposed mechanisms support the potential utility of the serum level of HFABP as an effective biomarker in patients with AKI, particularly those with a Class I/F status, on admission.

However, using the serum HFABP level to detect patients with AKI would have several limitations in comparison with other reno-tubular biomarkers such as serum NGAL level or NAG level, as the elevation of these reno-tubular biomarkers in serum clearly indicates renal damage and has superior diagnostic and prognostic significance [6–11]. NGAL is a 25-kDa protein normally secreted by renal tubular cells, leukocytes, and several other types of epithelial cells in response to ischemic or toxic injury [33]. In patients with AKI, various stresses first increase the serum NGAL in the circulation, which induces the activation of neutrophils. Then, the increased amount of NGAL is filtered from the glomerular filtrate. Some NGAL molecules are reabsorbed by the damaged proximal tubules, while others are not. Increased urinary NGAL is therefore mainly due to impaired renal re-absorption [33], and it takes a relatively long period of time for the urinary NGAL levels to return to normal. NAG is a lysosomal enzyme which is abundantly present in the cells of the proximal kidney tubule, and it can also be used to detect renal injury [34]. HFABP was categorized as a cardiovascular marker, not a reno-tubular biomarker. While the serum level of HFABP may indeed be an effective biomarker for AKI in the cardiovascular field, it might not be as useful as the available reno-tubular biomarkers. The predictive performance and scientific/clinical impact might be improved by the combined measurement of NGAL and HFABP. Two new metrics, the integrated discrimination improvement (IDI) and net reclassification improvement (NRI) were effective for improving the diagnostic value by quantifying the added value of a biomarker to an existing test [35, 36]. The measurement of serum NGAL level has already been reported as a definitive biomarker of AKI in non-surgical intensive care patients [12–14]. Therefore, the diagnostic value of the serum HFABP level might be increased by adding the serum NGAL level to it using the IDI and NRI method. Further study will be required in order to improve the diagnostic value of AKI based on the results of present study.

### Study limitations

Several limitations associated with the present study warrant mention. First, we were unable to obtain the medical records for all patients and were obliged to use the MDRD equation for serum creatinine (CrMDRD), assuming a GFR of 75 mL/min/1.73 m^2^ in order to determine the baseline values. This might be a major limitation of this study; Pickering et al. reported that a measured rather than estimated value should be used for baseline creatinine values in epidemiologic studies of AKI [37]. However, given that some patients in the present study had their creatinine levels measured only a limited number of times for unclear reasons, we had no choice but to use the MDRD equation. Of note, a new equation for estimating the GFR, defined as the CKD epidemiology collaboration (CKD-EPI) equation, was recently proposed [38]. This equation is reportedly more accurate than the MDRD study equation [38, 39]. However, although we tried to innovate this equation, race-based modifications were needed. While a modified CKD-EPI for use in Japanese populations has been reported [40], there is still no definitive equation for Asian populations. We therefore decided against using the CKD-EPI equation in this study. Second, some of the urinary and serum biomarkers, such as the urinary excretion of NGAL, LFABP, and NAG, could not be evaluated. These untested biomarkers may more clearly demonstrate the presence of AKI than the serum level of HFABP. Third, this study was performed at a single center and included a small number of subjects. The etiology was mixed, and the number of patients with each disease may have been too small to accurately evaluate the incidence of AKI. Fourth, diagnosing CKD based on the lowest value recorded in the year before admission is important; however, this was difficult to achieve in all patients in the present study. The creatinine level before admission was not measured in several patients (29.7 %), and the diagnosis of CKD was made based on previous medical records in 67 of 211 patients (31.8 %). Finally, the technique for measuring the H-FABP level was changed in July 2012 by the DS Pharma Biomedica. We therefore changed our measurement method from a MARKIT-M HFABP enzyme-linked immunosorbent assay kit to a LIBLIA H-FABP latex agglutination turbidimetric immunoassay. This use of a different method was another major limitation in the present study; however, the results from each method correlated well (*r* = 0.994). As such, we feel that the values of HFABP obtained via these different methods were comparable.

## Conclusions

The serum HFABP level is an effective biomarker for detecting AKI, especially a Class I/F status, on admission and produces an optimum balance between sensitivity and specificity for indicating the presence of AKI and a Class I/F status in non-surgical intensive care patients.

## Abbreviations

AHF: Acute heart failure
AKI: Acute kidney injury
AUC: Area under the ROC curve
BP: Blood pressure
CKD: Chronic kidney disease
GFR: Glomerular filtration rate
HFABP: Heart-type fatty acid-binding protein
HR: Hazard ratio
hs-TropT: High-sensitivity troponin-T
ICU: Intensive care unit
LFABP: Liver fatty acid-binding protein
MDRD: Modification of diet in renal disease
NAG: N acetyl-β-D glucosaminidase
NGAL: Neutrophil gelatinase-associated lipocalin
Nt-proBNP: N-terminal pro-brain-type natriuretic peptide
RIFLE criteria: The risk, injury, failure, loss and end stage criteria
ROC curve: Receiver-operating characteristic curve

## References

[1] Bellomo R, Kellum JA, Ronco C (2007). Defining and classifying acute renal failure: from advocacy to consensus and validation of the RIFLE criteria. Intensive Care Med.

[2] Bellomo R, Ronco C, Kellum JA, Mehta RL, Palevsky P, Acute Dialysis Quality Initiative workgroup (2004). Acute renal failure- definition, outcome measures, animal models, fluid therapy and information technology needs; the Second International Consensus Conference of the Acute Dialysis Quality Initiative (ADQI) Group. Crit Care.

[3] Hata N, Yokoyama S, Shinada T, Kobayashi N, Shirakabe A, Tomita K (2010). Acute Kidney injury and outcomes in acute decompensated heart failure: evaluation of the RIFLE criteria in an acutely ill heart failure population. Eur J Heart Fail.

[4] Shirakabe A, Hata N, Kobayashi N, Shinada T, Tomita K, Tsurumi M (2012). Long-term prognostic impact after acute kidney injury in patients with acute heart failure: evaluation of the RIFLE criteria. Int Heart J.

[5] Shirakabe A, Hata N, Kobayashi N, Shinada T, Tomita K, Tsurumi M (2013). Prognostic impact of acute kidney injury in patients with acute decompensated heart failure. Circ J.

[6] Ferguson MA, Vaidya VS, Waikar SS, Collings FB, Sunderland KE, Gioules CJ (2010). Urinary liver-type fatty acid-binding protein predicts adverse outcomes in acute kidney injury. Kidney Int.

[7] Doi K, Negishi K, Ishizu T, Katagiri D, Fujita T, Matsubara T (2011). Evaluation of new acute kidney injury biomarkers in a mixed intensive care unit. Crit Care Med.

[8] Matsui K, Kamijo-Ikemori A, Sugaya T, Yasuda T, Kimura K (2012). Usefulness of urinary biomarkers in early detection of acute kidney injury after cardiac surgery in adults. Circ J.

[9] Katagiri D, Doi K, Honda K, Negishi K, Fujita T, Hisagi M (2012). Combination of two urinary biomarkers predicts acute kidney injury after adult cardiac surgery. Ann Thorac Surg.

[10] Manabe K, Kamihata H, Motohiro M, Senoo T, Yoshida S, Iwasaka T (2012). Urinary liver-type fatty acid-binding protein level as a predictive biomarker of contrast-induced acute kidney injury. Eur J Clin Invest.

[11] Liangos O, Perianayagam MC, Vaidya VS, Han WK, Wald R, Tighiouart H (2007). Urinary N-acetyl-beta-(D)-glucosaminidase activity and kidney injury molecule-1 level are associated with adverse outcomes in acute renal failure. J Am Soc Nephrol.

[12] Clerico A, Galli C, Fortunato A, Ronco C (2012). Neutrophil gelatinase-associated lipocalin (NGAL) as biomarker of acute kidney injury: a review of the laboratory characteristics and clinical evidences. Clin Chem Lab Med.

[13] Hjortrup PB, Haase N, Wetterslev M, Perner A (2013). Clinical review: Predictive value of neutrophil gelatinase-associated lipocalin for acute kidney injury in intensive care patients. Crit Care.

[14] Zhang A, Cai Y, Wang PF, Qu JN, Luo ZC, Chen XD (2016). Diagnosis and prognosis of neutrophil gelatinase-associated lipocalin for acute kidney injury with sepsis: a systematic review and meta-analysis. Crit Care.

[15] Mathew TH, Johnson DW, Jones GR (2007). Chronic kidney disease and automatic reporting of estimated glomerular filtration rate: revised recommendations. Med J Aust.

[16] Levey AS, Bosch JP, Lewis JB, Greene T, Rogers N, Roth D (1999). A more accurate method to estimate glomerular filtration rate from serum creatinine: a new prediction equation. Modification of Diet in Renal Disease Study Group. Ann Intern Med.

[17] Iseki K (2008). Chronic kidney disease in Japan. Intern Med.

[18] Niizeki T, Takeishi Y, Arimoto T, Takabatake N, Nozaki N, Hirono O (2007). Heart-type fatty acid-binding protein is more sensitive than troponin T to detect the ongoing myocardial damage in chronic heart failure patients. J Card Fail.

[19] Inoue K, Suwa S, Ohta H, Itoh S, Maruyama S, Masuda N (2011). Heart fatty acid-binding protein offers similar diagnostic performance to high-sensitivity troponin T in emergency room patients presenting with chest pain. Circ J.

[20] Ecollan P, Collet JP, Boon G, Tanguy ML, Fievet ML, Haas R (2007). Pre-hospital detection of acute myocardial infarction with ultra-rapid human fatty acid-binding protein (H-FABP) immunoassay. Int J Cardiol.

[21] Seino Y, Tomita Y, Takano T, Ohbayashi K, Tokyo Rapid-Test Office Cardiologists (Tokyo-ROC) Study (2004). Office cardiologists cooperative study on whole blood rapid panel tests in patients with suspicious acute myocardial infarction: comparison between heart-type fatty acid-binding protein and troponin T tests. Circ J.

[22] Reiter M, Twerenbold R, Reichlin T, Mueller M, Hoeller R, Moehring B (2013). Heart-type fatty acid-binding protein in the early diagnosis of acute myocardial infarction. Heart.

[23] Viswanathan K, Kilcullen N, Morrell C, Thistlethwaite SJ, Sivananthan MU, Hassan TB (2010). Heart-type fatty acid-binding protein predicts long-term mortality and re-infarction in consecutive patients with suspected coronary syndrome who are troponin-nagative. J Am Coll Cardiol.

[24] Goto T, Takase H, Toriyama T, Sugiura T, Sato K, Ueda R (2003). Circulating concentrations of cardiac proteins indicate the severity of congestive heart failure. Heart.

[25] Seino Y, Ogata K, Takano T, Ishii J, Hishida H, Morita H (2003). Use of a whole blood rapid panel test for heart-type fatty acid binding protein in patients with acute chest pain: comparison with rapid troponin T and myoglobrin tests. Am J Med.

[26] Setsuta K, Seino Y, Ogata T, Arao M, Miyatake Y, Takano T (2002). Use of cytosolic and myofibril markers in the detection of ongoing myocardial damage in patients with chronic heart failure. Am J Med.

[27] Hazui H, Negoro N, Nishimoto M, Muraoka H, Murai M, Takeshita H (2005). Serum heart-type fatty acid-binding protein concentration positively correlates with the length of aortic dissection. Circ J.

[28] Dellas C, Puls M, Lankeit M, Schäfer K, Cuny M, Berner M (2010). Elevated heart-type fatty acid-binding protein levels on admission predict an adverse outcome in normotensive patients with acute pulmonary embolism. J Am Coll Cardiol.

[29] Boscheri A, Wunderlich C, Langer M, Schoen S, Wiedemann B, Stolte D (2010). Correlation of heart-type acid-binding protein with mortality and echocardiogenic data in patients with pulmonary embolism at intermediate risk. Am Heart J.

[30] Jo YH, Kim K, Lee JH, Rhee JE, Lee JH, Kang KW (2012). Heart-type fatty acid-binding protein as a prognostic factor in patients with severe sepsis and septic shock. Am J Emerg Med.

[31] Tanaka T, Hirota Y, Sohmiya K, Nishimura S, Kawamura K (1991). Serum and urinary human heart fatty acid-binding protein in acute myocardial infarction. Clin Biochem.

[32] Yan GT, Lin J, Hao XH, Xue H, Zhang K, Wang LH (2009). Heart-type fatty acid-binding protein is a useful marker for organ dysfunction and leptin alleviate sepsis-induced organ injuries by restraining its tissue levels. Eur J Pharmacol.

[33] Mori K, Lee HT, Rapoport D, Drexler IR, Foster K, Yang J (2005). Endocytic delivery of lipocalin-siderophore-iron complex rescues the kidney from ischemia-reperfusion injury. J Clin Invest.

[34] Skálová S (2005). The diagnostic role of urinary N-acetyl-beta-D-glucosaminidase (NAG) activity in the detection of renal tubular impairment. Acta Med (Hradec Kralove).

[35] Pickering JW, Endre ZH (2012). New metrics for assessing diagnostic potential of candidate biomarkers. Clin J Am Soc Nephrol.

[36] Parikh CR, Thiessen-Philbrook H (2014). Key concepts and limitations of statistical methods for evaluating biomarkers of kidney disease. J Am Soc Nephrol.

[37] Pickering JW, Endre ZH (2010). Back-calculating baseline creatinine with MDRD misclassifies acute kidney injury in the intensive care unit. Clin J Am Soc Nephrol.

[38] Levey AS, Stevens LA, Schmid CH, Zhang YL, Castro AF, Feldman HI (2009). A new equation to estimate glomerular filtration rate. Ann Intern Med.

[39] Stevens PE, Levin A, Kidney Disease: Improving Global Outcomes Chronic Kidney Disease Guideline Development Work Group Members (2013). Evaluation and management of chronic kidney disease: synopsis of the kidney disease: improving global outcomes 2012 clinical practice guideline. Ann Intern Med.

[40] Horio M, Imai E, Yasuda Y, Watanabe T, Matsuo S (2010). Modification of the CKD epidemiology collaboration (CKD-EPI) equation for Japanese: accuracy and use for population estimates. Am J Kidney Dis.

